# Selenium Derivatization of Nucleic Acids for Phase and Structure Determination in Nucleic Acid X-ray Crystallography

**DOI:** 10.3390/ijms9030258

**Published:** 2008-03-12

**Authors:** Jia Sheng, Zhen Huang

**Affiliations:** Department of Chemistry, Georgia State University, Atlanta, GA 30303, USA

**Keywords:** Selenium, nucleic acid, X-ray crystallography, phase determination

## Abstract

Selenium derivatization (*via* selenomethionine) of proteins for crystal structure determination via MAD phasing has revolutionized protein X-ray crystallography. It is estimated that over two thirds of all new crystal structures of proteins have been determined via Se-Met derivatization. Similarly, selenium functionalities have also been successfully incorporated into nucleic acids to facilitate their structure studies and it has been proved that this Se-derivatization has advantages over halogen strategy, which was usually used as a traditional method in this field. This review reports the development of site-specific selenium derivatization of nucleic acids for phase determination since the year of 2001 (mainly focus on the 2’-position of the ribose). All the synthesis of 2’-SeMe modified phosphoramidite building blocks (U, C, T, A, G) and the according oligonucleotides are included. In addition, several structures of selenium contained nucleic acid are also described in this paper.

## 1. Introduction

Determination of three-dimensional structures of nucleic acids and protein-nucleic acid complexes is important for gaining understanding of the biological systems. It can provide fundamental insights into transcription and translation regulation, DNA replication mechanisms, DNA-drug complexes, catalytic RNAs, riboswitch functions, and nucleic acid-protein interactions [1–3]. The major methods for macromolecular 3D-structure study include X-ray crystallography, NMR, electron microscopy, and atomic force microscopy [4]. Among these powerful tools, X-ray crystallography is one of the best strategies for high-resolution structure determination [5–7]. Due to technology advancements, crystallography has allowed the 3D structure determination of a tremendous amount of nucleic acids as well as their protein complexes over the last ten years [5–8]. However, there are still two major limiting factors: crystallization and phase problem, which prevents crystallography from fully realizing its potential in this application [5–7,9,10]. So far, there are no rational approaches to crystallize these macromolecules, and many factors can affect the crystal growth. Crystallization is usually achieved through a trial-and-error screening strategy. Another most serious bottleneck in macromolecule crystallography is the phase problem, which has also largely slowed down structural determination of new nucleic acid folds and the protein complexes. Phase information is needed to calculate 3D structure of macromolecules after collected diffraction data.

Conventionally, the phase problem can be solved by the so-called multiple isomorphous replacement method (MIR), which requires at least three isomorphous crystals, including two heavy atom derivatives. Iodine derivatization of nucleic acids has been used for this purpose. Heavy metal soaking and co-crystallization are also normally used to prepare the derivatized crystals [11]. Unlike protein crystal derivatization, the soaking and co-crystallization do not work well with nucleic acids probably because nucleic acids are polyanion species and often lack specific binding sites for metal ions [12]. Furthermore, new technologies for solving the phase problem, such as multiple- or single-wavelength anomalous dispersion (MAD or SAD), have been developed to take advantage of anomalous scattering properties of some heavy atoms (e.g., bromine and selenium) [13]. These methods allow collection of entire diffraction data set for structure determination from a single crystal. The bromine derivatization of nucleic acids has been used as a conventional strategy for phase determination via MAD phasing [14,15]. However, the limited positioning for the Br derivatization, normally at the 5-positions of pyrimidine nucleosides, can cause base-stacking disruption, groove perturbation, changes in hydration patterns, and even crystallization problems. Moreover, recent reports have indicated that light-sensitive halogen derivatives are also sensitive to X-ray radiation during diffraction data collection, causing molecule and crystal decomposition [16].

Selenium derivatization (with selenomethionine) of proteins for crystal structure determination via MAD phasing has revolutionized protein X-ray crystallography [5,7,17,18], and it is estimated that over two thirds of all new crystal structures of proteins have been determined via Se-Met derivatization [5,19,20]. Successful protein X-ray crystallography via the selenomethionine derivatization and nucleic acid indirect derivatization with Se-labeled proteins [10,21] inspired us to directly incorporate selenium covalently into nucleic acids for phase and structure determination of nucleic acids and their protein complexes. Since 1998, our research group has pioneered selenium derivation of nucleic acids for X-ray crystallographic studies [22,23]. So far, we have successfully developed various chemical and enzymatic strategies to atom-specifically substitute oxygen with selenium and covalently incorporate Se-functionality into nucleic acids at a variety of positions, including 5’, 2’, nucleobase and phosphate non-bridging positions [15,22–31]. Through collaboration among Huang, Egli and co-workers, we have determined for the first time structure of a nucleic acid covalently-derivatized with selenium via MAD phasing [32]. This atom-specific derivatization with selenium leads to synthesis of selenium-derivatized nucleic acids (SeNA), which have great potential in structural and functional studies of nucleic acids. So far, this novel derivatization strategy has been utilized in X-ray crystal structure studies of RNA and DNA molecules by us and several other laboratories [15,30–37], including structure analysis and determination of ribozymes, riboswitches and homo-DNA. More excitingly, we have recently determined crystal structures of nucleic acid-protein complexes via the SeNA derivatization instead of the protein selenomethionine derivatization [38]. SeNA synthesis and purification are much easier than Se-protein expression and purification. Therefore, we have opened a novel research avenue for structure and function studies of protein-nucleic acid complexes.

**Scheme 1.**
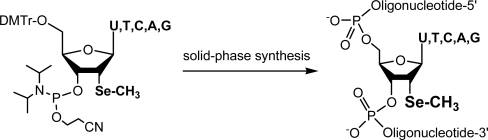
Structures of 2’-Se-nucleoside phosphoramidites and SeNAs. C, A and G nucleobases in phosphoramidites are protected by acyl groups.

Our first attempt in nucleic acid Se-derivatization involved the incorporation of Se to the C5’-positions of A, C, G and T by replacing the oxygen with selenium [22]. Importantly, it proved that the selenium functionality is compatible with automated solid phase synthesis. Furthermore, we introduced a methylseleno group to the α-2’-position of uridine (2’-SeMe-U), and synthesized DNAs and RNAs derivatized with 2’-Se-derivatization for the first time [23]. Through the collaboration, we also determined the first Se-derivatized nucleic acid structure by Se MAD phasing [32]. Later, other research groups reported additional SeNA synthesis and crystal structure studies via the selenium derivatization [35,39]. As shown by us and other groups, this site-specific selenium derivatization strategy is a better alternative over the halogen derivatization in stability, crystal diffraction quality, and derivatization positioning [15,30,31]. As selenium and oxygen are in the same element family VI in the periodic table, replacement of oxygen with selenium may not cause a significant structural perturbation when Se is placed at an appropriate position. There are numerous oxygen atoms available in nucleic acids, thereby providing more positioning options for selenium replacement of oxygen. Among these selenium replacements, the 2’-Se-modifications are most stable and convenient to use, this review briefly summarizes the synthesis of all 2’-Se-modified U, T, C, A and G building blocks (Scheme 1), and their applications in structure determination and function study.

## 2. Synthesis and application of 2’-Se-pyrimidines

**Scheme 2.**
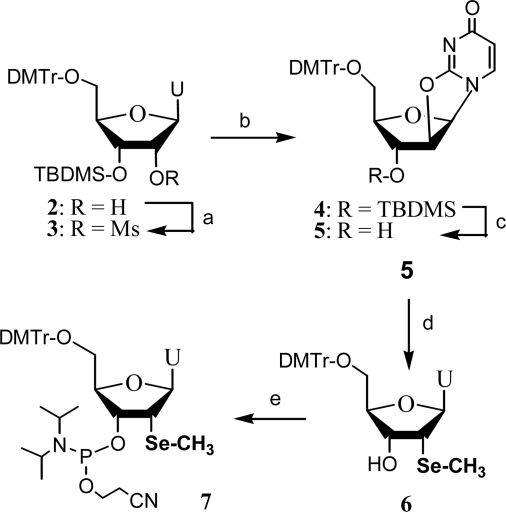
Synthesis of 2’-methylseleno contained uridine phosphoramidite and its incorporation into oligonucleotides. (a) MsCl/THF/TEA, 95% yield; (b) toluene/tetrahexylammonium hydrogen sulfate/Na_2_CO_3_ (sat.), 96% yield; (c) (Bu)_4_N^+^ F^−^, 95% yield; (d) NaHSe, then CH_3_I, or NaSeCH_3_, 96%; (e) PCl(OCH_2_CH_2_CN)N(iPr)_2_, 92% yield.

As a pioneering trial in this methodology [23], the synthesis of 2’-Se-uridine phosphoramidite and its containing oligonucleotides were achieved through the 5’-protected-2,2’-anhydrouridine as intermediate, which was generated by the replacement of 2’-mesyl group by the uracil exo-2-oxygen under a two-phase reaction system catalyzed by phase-transfer catalyst (Scheme 2, step b). The 3’-bulky TBDMS group was removed with fluoride treatment since it blocked the subsequent selenide nucleophile attacking from the 2’-α-face. We initially tried to introduce the hydroseleno group using NaHSe generated by reduction of selenium metal with NaBH_4_. However, the resulting selenol compound can be rapidly oxidized when it is exposed to air. This problem was resolved by protection of the selenium atom with a methyl group or using NaSeMe as the nucleophile. The selenium containing compound **6** was confirmed by MS, ^77^Se-NMR, 2D-NMR and NOE, followed by its conversion to the phosphoramidite **7**. Using this building block, several DNA and RNA analogues containing this 2’-SeMe functionality were synthesized following standard solid phase synthesis procedures, even on a 10-micromol scale. As expected, the selenium functionality was found to be stable towards mild I_2_ treatment, and all the oligonucleotides could be purified by standard RP-HPLC. In terms of thermal stability, it was found that even incorporation of two dU_Se_ residues in a self-complementary sequence had no effect to the Tm comparing with native controls. We have achieved incorporation of 7 selenium atoms into one DNA with 32 nt [25,26].

**Figure 1.**
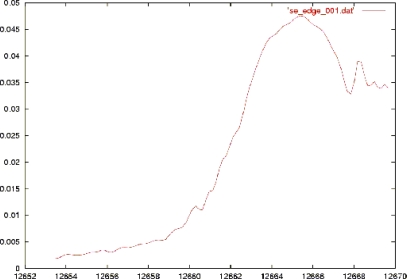
X-ray fluorescence spectrum of the decamer crystal. The theoretical value for the Se K edge is 12.6578 keV (0.9795 Å).

To further check the anomalous scattering power of selenium, the crystal structure of a Se-DNA decamer duplex [GCGTA(2’-Se-U)ACGC)]_2_ was also investigated in a high resolution of 1.3 Ǻ in which the diffraction data was successfully phased on the basis of selenium anomalous signal [32]. This structural data showed the 2’-methylseleno group was directed into the minor groove and the C3’-C2’-Se-Me torsion angle adopt an antiperiplanar conformation. In addition, this modified furanose displayed the C3’-endo sugar pucker, consistent with the A-form of DNA and RNA. Figure 1 shows the absorption of selenium atom in X-ray fluorescence spectrum and in this experiment, three sets of data were collected for MAD: the inflection point (edge), the absorption maximum (peak) and approximately 460 eV above the peak wavelength (remote).

**Figure 2.**
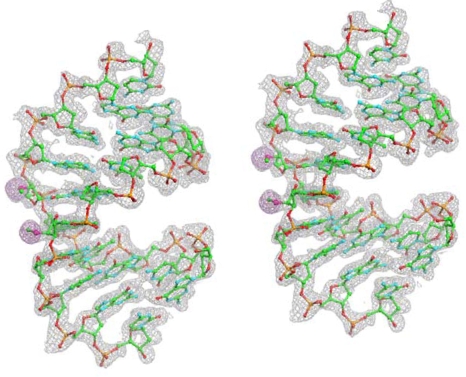
Fourier electron density maps for the final structure of the decamer DNA duplex [GCGTA(2’-Se-U)ACGC)]_2_ calculated at 1.3 Å resolution with MAD phases. DNA atoms are colored green, cyan, red and orange for carbon, nitrogen, oxygen and phosphorus, respectively, and the two selenium atoms per asymmetric unit are highlighted in pink.

It is also worth pointing out that the data collected at the peak wavelength is sufficient for SAD phasing when high-resolution diffraction data are collected. The Fourier electron density maps were computed (Figure 2) based on the refinement of the two selenium positions. From the determined structure, the hydration pattern surrounding the selenium atoms was disclosed, which shows that the higher hydrophobicity of selenium than oxygen or sulfur is of little consequence in terms of the number of surrounding water molecules. This introduction of selenium at the sugar 2’-position did not affect the minor groove hydration significantly.

**Scheme 3.**
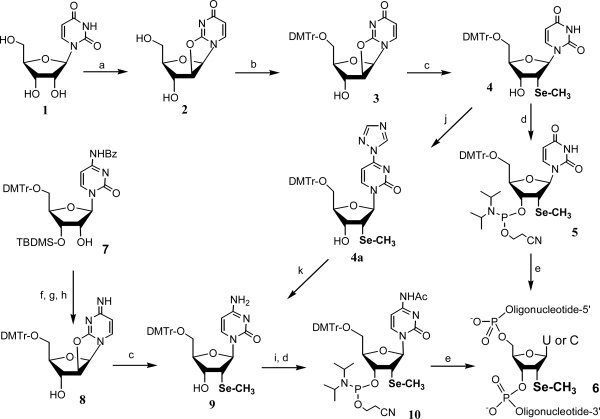
Improved synthesis of 2’-methylseleno contained uridine and cytidine phosphoramidite and their incorporation into oligonucleotides. (a) (Ph)_2_CO_3_, Na_2_CO_3_, DMF; (b) DMTr-Cl, Py; (c) NaSeCH_3_, EtOH-THF; (d) 2-cyanoethyl N,N-diisopropyl-chlorophosphoramidite and N,N-diisopropylethylamine in CH_2_Cl_2_; (e) synthesis of oligonucleotides on solid phase; (f) Ms-Cl, TEA, THF; (g) toluene/tetrahexylammonium hydrogen sulfate, Na_2_CO_3_ (satd); (h) (Bu)_4_N^+^ F^−^, THF; (i) TMS-Im, then Ac_2_O, TEA and DMAP in THF; (j) TMS-Im, then POCl_3_-triazole-TEA in CH_3_CN; (k) NH_4_OH.

Encouraged by the structure data, we developed an improved synthetic route for efficient incorporation of the selenium functionality and for synthesis of both the Se-uridine and Se-cytidine phosphoramidites (Scheme 3) [24,25]. This synthesis started from relatively inexpensive uridine (**1**), and the 2,2’-anhydrouridine intermediate **3** could be made on a 50-gram scale in high yield. In addition, after the incorporation of the 2’-selenomethyl group, the resulting uridine derivative can also be converted to a cytidine analog via triazolide activation, followed by ammonia treatment. Since the strategy of synthesizing the selenocytidine (from **8** in Scheme 3) did not work well, this method of converting U to C became a useful synthesis to produce large amounts of the 2’-Se-cytidine phosphoramidite. In the Se-RNA synthesis, a coupling reagent 5-BMT [5-(benzylmerapto)-1H-tetrazole] was used in combination with 2’-TOM protection to synthesize several Se-RNAs with different secondary structures in high yields [24,25]. Moreover, the thermal denaturization and crystallization of the self-complementary Se-octamer duplex [G(2’-Se-U)GTACAC]_2_ were also studied and compared with the corresponding native structures. Consistent with the previous structural results, the dUSeMe residues have no significant effect on duplex stability of the A-form DNA. More excitingly, we found that this Se-DNA is easy to crystallize in many more screen conditions than the corresponding native and Br-modified DNAs.

**Figure 3.**
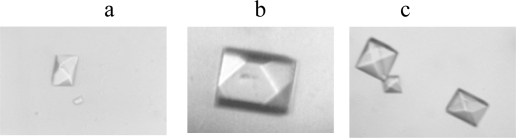
Photos of crystals of the native and Se-derivatized octamers. (a) Native-Oct.; (b) Se-Oct.; (c) Se/Br-Oct. Sizes of the crystals are ranged from 0.1×0.1 mm to 0.4×0.4 mm.

Very recently, the high resolution structure of this DNA [G(2’-Se-dU)GTACAC]_2_ was determined via MAD and SAD technology at 1.28 Ǻ resolution [15]. To directly compare this selenium strategy with conventional bromine strategy, the same sequence with bromine derivatization [GUG(5-Br-dU)ACAC] and the one with both selenium and bromine [G(2’-Se-dU)G(5-Br-dU)ACAC] were synthesized. As shown in Figure 3, the morphology of Se and Br derivatized crystals seemed very similar. Furthermore, their X-ray structures were obtained at 1.8 Ǻ and 1.5 Ǻ resolution, respectively. Selenium atoms were located in the minor groove, and no structural perturbation has been observed in Se-DNA global and local structures, comparing with the native A-form DNA. The structural data for Br-DNA and Se-Br-DNA showed that there were no obvious global structural perturbations, compared to the native one. However, a large local perturbation caused by the bromine derivatization was observed, including a sugar pucker change and an approximately 108-degree rotation about its C4’-C5’ bond. Moreover, the hydration pattern and the water networking at the Br-derivatized site were also altered significantly. These may explain why sometimes Br-derivatized DNAs are more difficult to crystallize than their native counterparts, or they cannot crystallize under the native conditions.

**Figure 4.**
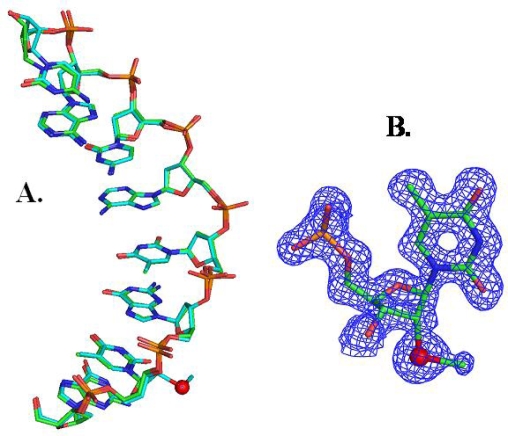
DNA crystal structures (Se in red; only single strand is shown here). (A) Superimposed structure comparison of the native DNA (GTGTACAC, 1DNS in green) and the Se-derivatized DNA (GTSeGTACAC, 2HC7 in cyan). (B) The structure model and the electron density map of 2’-Seribothymidine in the structure.

Besides its usefulness in structural study, we also observed that this type of selenium derivatization can greatly facilitate the crystallization process. In case of the Se-DNA [G(2’-Se-dU)GTACAC]_2_, all of the 24 Hampton mini-screen buffers work well, and the crystals could grow overnight. On contrary, the native and Br-derivatized DNAs needed 2–3 months to grow. More interestingly, this observation was not only for this sequence, but also for several other DNA sequences containing 2’-Se at different positions, such as positions close to 5’, 3’, or the middle region. Comparing with the native counterparts, the selenium modified oligonucleotides can crystallize under much broader buffer conditions. Similarly to the method for the Se-uridine synthesis, the 2’-Se-thymidine phosphoramidite was synthesized due to the consideration that ribothymidine (rT) is a common modification in mature tRNAs and rRNAs [30]. Selenium derivatization at the 2’-position in these RNAs helps not only to determine their structures at higher resolutions, but also to obtain new insight into the importance of the rT modifications and functions in natural RNAs. After 2’-Se-rT incorporation into several DNAs and RNAs (including the TψC loop) with high yields, we successfully obtained the crystal structure of G(2’-Se-T)GTACAC and showed that there is no significant perturbation compared to the native one (Figure 4). This structure result is consistent with the thermodenaturization study. In addition, the same phenomenon that this 2’-selenium modification facilitates crystallization was also observed with 2’-Se-rT.

## 3. Synthesis and application of 2’-Se-purines

**Scheme 4.**
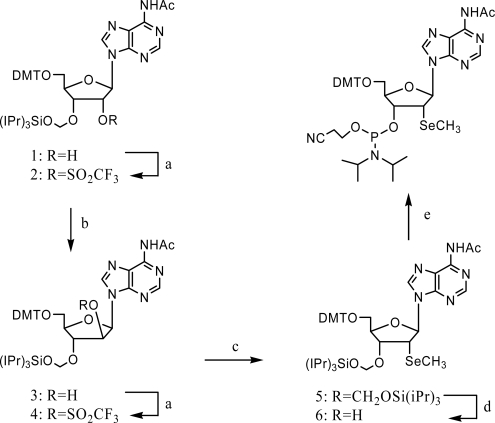
Synthesis of the 2’-Se-methyladenosine phosphoramidite. Reagents and conditions: (a) 1.5 equiv. trifluoromethanesulfonyl chloride, 1.5 equiv DMAP, 2.5 equiv NEt_3_, in CH_2_Cl_2_, room temperature, 20 min; (b) 8.3 equiv CH_3_COO^−^K^+^, 2.5 equiv. (iPr)_2_NEt, 3.3 equiv. 18-crown-6-ether, in toluene, 80 °C, 11 h [54% over (a) and (b)]; (c) 6 equiv. NaBH_4_, 2 equiv. CH_3_SeSeCH_3_, in THF, 30min; (d) 1 M TBAF, 0.5 M acetic acid, in THF, r.t. 2.5 h, 84%. (e) 1.5 equiv. (2-cyanoethyl)-N,N-diisopropylchlorophosphoramidite, 10 equiv. CH_3_CH_2_N(CH_3_)_2_, CH_2_Cl_2_, r.t, 2 h, 91%. (DMT: dimethoxytrityl, DMAP: 4-(dimethylamino)pyridine; TBAF (tetrabutylammonium fluoride).

Due to this promising selenium strategy for nucleic acid crystallography, the synthesis of 2’-Se-purine phosphoramidites was achieved by Micura and co-workers. A good leaving group, such as Tf (trifluoromethanesulfonyl), was introduced to the 2’-β-position, followed by sodium methylselenide substitution. This method led to the successful synthesis of 2’-methylseleno-adenosine and the 2’-SeA-RNAs (Scheme 4) [39]. In this work, an additional DTT treatment step was inserted into the RNA solid phase synthesis, to prevent the potential oxidation of selenium atoms, which would result in a big difficulty for the purification step and reduction of the yields. As a result, it was suggested that DTT was necessary for the reliable synthesis of RNAs (>25 nt) especially containing multiple Se labels during their synthesis, deprotection and crystallization. Based on that, eleven functional RNA oligonucleotides containing different numbers and types of selenium (up to seven) residues were synthesized including the Diels-Alder ribozyme and the aptamer domain of the adenine deaminase (add) adenine-riboswitch.

A Se-RNA crystallization was carried out for a 49 nt Diels-Alder ribozyme [35], which could accelerate the Diels-Alder cyclization reactions with high enentioselectivity. Interestingly, the crystallization conditions of the selenium modified RNA didn’t change, compared to the native ones, which meant that this 2’-Se derivatization didn’t affect the double helix formation by locating in the minor groove. The well-solved structures in both unbound and bound states with the reaction product have provided a detailed insight into its catalytic pocket and a possible mechanism of carbon-carbon formation based on that the stereoselectivity could be achieved.

**Scheme 5.**
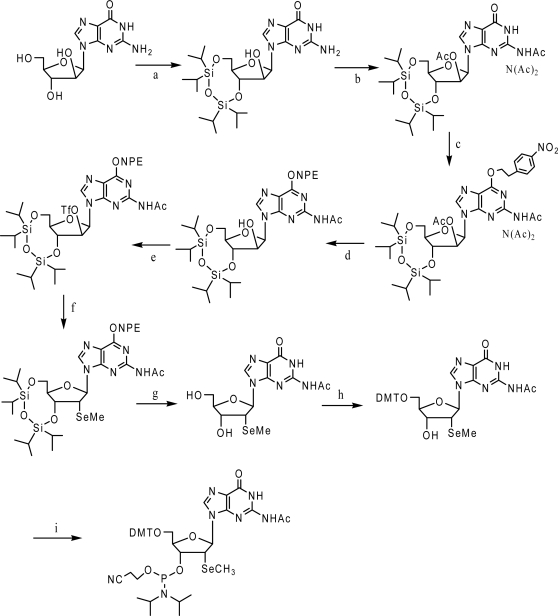
Synthesis of the 2’-methylseleno guanosine phosphoramidite. (a) 1.05 equiv of TIPDSiCl_2_, in DMF/pyridine, room temperature, 16 h, 98% (b) 10 equiv. of acetic anhydride, in DMF/pyridine, 80 °C, 16 h, 64% (c) 1.3 equiv. of NPE-OH, 1.4 equiv. of PPh3, 1.3 equiv. of DIAD, in dioxane, room temperature, 3 h, 64% (d) aqueous ammonium hydroxide in THF/methanol/water, 0 °C, 5 min, 57% (e) 1.5 equiv. of trifluoromethanesulfonyl chloride, 1.5 equiv. of DMAP, 2.5 equiv. of NEt_3_, in CH_2_Cl_2_, 0 °C, 2 h, 69% (f) 6 equiv. of NaBH_4_, 2 equiv. of CH_3_SeSeCH_3_, in THF, 20 min, 87% (g) 1 M TBAF, in THF, room temperature, 2.5 h, 79% (h) 1.1 equiv. of DMT-Cl, in pyridine, room temperature, 16 h, 59% (i) 1.5 equiv. of (2-cyanoethyl)-N,N-diisopropylchlorophosphoramidite, 10 equiv of CH_3_CH_2_N(CH_3_)_2_, in CH_2_Cl_2_, room temperature, 2 h, 88% (DIAD diisopropyl azodicarboxylate, DMAP 4-(dimethylamino)-pyridine, DMT dimethoxytrityl, NPE 2-(4-nitrophenyl)ethyl, TBAF tetrabutylammonium fluoride, TIPDSiCl_2_ 1,3-dichloro-1,1,3,3-tetraisopropyldisiloxane).

The Se-guanosine phosphoramidite was also synthesized (Scheme 5) [36]. The general synthesis was similar to the 2’-SeMe-A synthesis, except for the mandatory protection of O-6 position since it will react with the trifluoromethanesulfonyl chloride reagent and the optimized hydrolysis step to liberate the arabinose 2’-hydroxyl group while retain the guanine N2 monoacetyl group (Scheme 5, c and d). This building block was incorporated into oligonucleotides under the modified synthesis including the DTT treatment. Furthermore, the redox behavior of selenium atom was investigated. The 2’-methylseleno group may be oxidized to methylselenoxide under the iodine treatment, which can be converted back to the methylseleno under reductive conditions like DTT treatment.

## 4. Facilitation of DNA Crystallization by Selenium Derivatization

In our crystallization study, we found that the Se-DNA [G(2’-Se-T)GTACAC] crystallized in two to three weeks from the native buffer, where the native DNA (GTGTACAC) crystallized over two months [15,30]. When 24 diversified random crystallization buffers were used for screening, to our pleasant surprise, we found that the Se-DNA crystallized overnight in 20 out of 24 buffers. In contrast, the native DNA did not crystallize at all after many weeks in these buffers. Interestingly, the two RNAs (12mer and 16mer) containing the 2’-Se-guanosines crystallized respectively in 17 and 33 buffers [36], while their corresponding native RNAs crystallized in 15 and 24 buffers, individually. Though the crystallization conditions of these Se-RNAs expanded slightly, it appears that these two Se-RNAs (12mer and 16mer), which crystallized mostly as thin needle crystals, behaved similarly as many Se-Met-derivatized proteins that required the fine-tuning of their native crystallization conditions. By contrast, the majority of these Se-DNA crystals grown under the new buffers were larger than the native DNA crystals grown under the native buffer conditions, and these Se-DNA crystals also diffracted well. Furthermore, many other DNA sequences were tested [15], and the same phenomena of crystallization facilitation was observed. Our experimental results reveal that the 2’-Se derivatization facilitates the crystal packing and high-quality crystal growth, and expands the crystallization conditions.

## 5. Prospects of Selenium Derivatization of Nucleic Acids

This site-specific 2’-Se-derivatization strategy has many obvious advantages in nucleic acid X-ray crystallography via SAD and MAD phasing. Unlike the uncontrollable heavy atom soaking and co-crystallization methods, Se derivatization can be easily used to incorporate selenium into atom-specific positions of DNAs and RNAs. Comparing with the Br derivatization method (with limited sites), Se has been incorporated into U, T, C, A, and G at various sites. In addition, unlike the bromine derivatization, this selenium derivatization is more stable during the X-ray exposure. More importantly, the structural data have already demonstrated that this minor groove-located Se-functionality will not cause significant structural perturbation. Moreover, this Se strategy may facilitate the nucleic acid crystal growth. Therefore, our novel discoveries on the selenium derivatization may provide rational strategies to both crystallization and phase determination. It is worth pointing out that due to the 2’-exo sugar pucker, this 2’-Se derivatization probably works better with A-DNAs and RNAs than B-DNA. For B-form DNA derivatization, we will continue to pursue the selenium derivatization on other positions, such as the nucleobase, phosphate, 3’, 4’ and 5’ positions [22,26,29,31]. Furthermore, the selenium-derivatization of nucleic acids can be used in structure and function studies of nucleic acid-protein complexes. We are confident that the selenium derivatization strategy will revolutionize X-ray crystallography of both nucleic acids and their protein complexes.
